# Scattered high-energy synchrotron radiation at the KARA visible-light diagnostic beamline

**DOI:** 10.1107/S1600577524001905

**Published:** 2024-03-26

**Authors:** David R. Batchelor, Edmund Blomley, Erhard Huttel, Michael Hagelstein, Akira Mochihashi, Marcel Schuh, Rolf Simon

**Affiliations:** a Karlsruhe Institute of Technology, Institute for Photon Science and Synchrotron Radaiation (IPS), Hermann-von-Helmholtz-Platz 1, D-76344 Eggenstein-Leopoldshafen, Germany; b Karlsruhe Institute of Technology, Institute for Beam Physics and Technology (IBPT), Hermann-von-Helmholtz-Platz 1, D-76344 Eggenstein-Leopoldshafen, Germany; University of Tokyo, Japan

**Keywords:** visible-light diagnostics, beamline, radiation shielding, high-energy synchrotron radiation, leakage dose

## Abstract

To characterize an electron beam, visible synchrotron light is often used and dedicated beamlines at synchrotron sources are becoming a more common feature as instruments and methods for the diagnostics are, along with the accelerators, further developed. At KARA (Karlsruhe Research Accelerator), such a beamline exists and is based on a typical infrared/visible-light configuration. From experience at such beamlines no significant radiation was expected (dose rates larger than 0.5 µSv h^−1^). This was found not to be the case and a higher dose was measured which fortunately could be shielded to an acceptable level with 0.3 mm of aluminium foil or 2.0 mm of Pyrex glass. The presence of this radiation led to further investigation by both experiment and calculation. A custom setup using a silicon drift detector for energy-dispersive spectroscopy (Ketek GmbH) and attenuation experiments showed the radiation to be predominantly copper *K*-shell fluorescence and is confirmed by calculation. The measurement of secondary radiation from scattering of synchrotron and other radiation, and its calculation, is important for radiation protection, and, although a lot of experience exists and methods for radiation protection are well established, changes in machine, beamlines and experiments mean a constant appraisal is needed.

## Abstract 2

High-energy scattered synchrotron radiation at the visible-light diagnostics beamline at KARA (Karlsruhe Research Accelerator) and consequences for the future design of beamlines and radiation shielding as accelerators, beamlines and methods/techniques develop are presented.

## 1. Introduction

The measurement of scattered secondary radiation from a synchrotron and its calculation is central to radiation protection. Although procedures are well established, changes in machine, new beamlines and experiments provide a constant impetus to improve and develop methods for measurement and calculation of the radiation. For example, the development of small-scale pulsed plasma-based accelerator sources and their related technology (Nasse *et al.*, 2013 ▸; Ghaith *et al.*, 2021 ▸; Bernhard *et al.*, 2018 ▸) is very different in the production, type of X-rays and energy range compared with the more traditional large-scale facilities (Einfeld *et al.*, 2000 ▸).

It is important for the operation of such sources that the electron beam parameters, and their control, responsible for the production of the light are accurately known. Visible light is frequently used and techniques (Boland *et al.*, 2012 ▸; Ikeda *et al.*, 2022 ▸) further developed along with the construction of dedicated beamlines for the measurements (Breunlin & Anderson, 2016 ▸; Panaś *et al.*, 2021 ▸; Schiwietz *et al.*, 2021 ▸). Such a beamline has been in use since 2011 (Hiller *et al.*, 2011 ▸) on a bending magnet at the Karlsruhe Reseach Accelerator (KARA) and is being further developed (Patil *et al.*, 2023 ▸).

**Figure 1:**
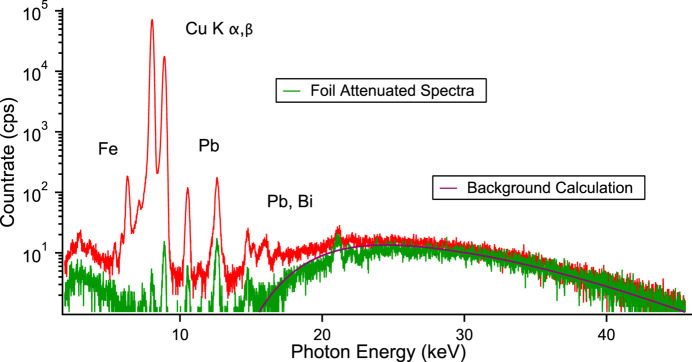
Experimental results of summed raw (red) and copper foil attenuated (green) SDD output. The purple curve is a result of a calculation of the scattered synchrotron radiation from the second copper mirror described in Section 3. Good agreement is seen between the calculated curve and the attenuated spectrum identifying the high-energy portion as high-energy scattered synchrotron radiation.

The reflecting optics are very similar to those at other infrared (Zheshen *et al.*, 2015 ▸) and UV beamlines (Bürck *et al.*, 2015 ▸), consisting of a chicane of 90° reflections. Dose rates at such beamlines are not high, below 0.5 µSv h^−1^. Surprisingly, a higher dose rate about two orders of magnitude higher than this value has been found. Fortunately, this radiation could be easily shielded to an acceptable level using 300 µm of aluminium foil or 2 mm of Pyrex glass, which suggested a relatively low energy of the radiation. Although the dose could be measured, the sensitivity of the dosimeters differed, and uncertainty as to the exact nature, spatial and energy distribution existed; the origin was not clear. Therefore it was decided to investigate this further by direct measurement using fluorescence detection and calculations. High-energy scattered synchrotron radiation was finally found to be responsible resulting in a large copper *K*-shell fluorescence reaching the diagnostics on the other side of the wall. The resulting fluorescence spectrum is shown in Fig. 1 ▸ (red curve) together with a copper foil attenuated spectrum (green curve) showing the high-energy synchrotron radiation background, and the results of a calculation (purple curve) of the background, described in the *Results and discussion* [Sec sec3] section.

The beamline layout and setup is discussed in the next section. Results of attenuation experiments together with calculations of the radiation dose and background are presented in Section 3 and compared with measurements. Finally, other typical scattering scenarios and resulting scattered radiation calculations are presented.

## 2. Experiment and method

**Figure 2:**
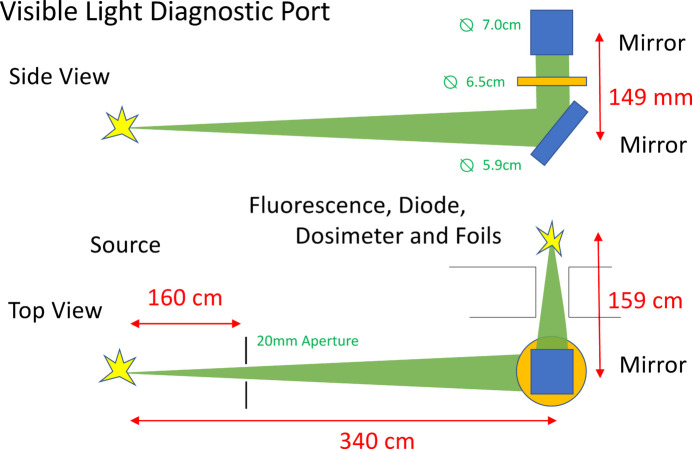
Schematic of the visible-light diagnostic port showing the hole in the radiation wall and the fluorescence detector, diode and dosimeter and attenuation foils setup (side and top views).

Fig. 2 ▸ shows a schematic of the beamline layout which is described in previous work (Hiller *et al.*, 2011 ▸). Synchrotron radiation passes through a 20 mm horizontal aperture and is captured by a water-cooled plane mirror (diameter 5.9 cm, solid angle 1.94 × 10^−5^ sterad) and is then deflected upwards by 90° through a 5 mm-thick UHV quartz window (diameter 6.5 cm) which isolates the vacuum and also acts as a filter transmitting the visible light and blocking X-rays. The visible light is then deflected sideways horizontally through a hole in the ring wall to the beam diagnostics by a second mirror (diameter 7.0 cm, solid angle 1.38 × 10^−2^ sterad). The second mirror is a polished copper parabolic mirror with a thin 30 µm reflecting aluminium coating. An aluminium profile frame was constructed to hold various interchangeable detectors. The fluorescence and scattered photons were measured with an energy-dispersive silicon drift detector (SDD) sensitive to radiation in the 2–40 keV range with an energy resolution of 135 eV (KETEK Gmbh, AXAS-M) and also measured using two non-dispersive detectors, a photodiode (Hamamatsu S3590-06) and a calibrated dosimeter (Berthold Tecnologies, Tol-F). The dosimeter measures the energy deposited in the detector by absorption of X-rays in an energy window from 10 keV to 7 MeV. The diode is very sensitive to visible and UV light which has to be blocked by a thin 25 µm black Kapton foil filter. The illuminated area of the dosimeter was 2 cm × 4 cm giving a solid angle of ∼2.5 × 10^−5^ sterad. The SDD output was processed using a digital signal processor and associated software (XIA LLC).

The diode, together with filter measurements, determined the extent of the radiation and showed that its effects would not be adverse for the fluorescence detector. Initial fluorescence measurements were made with a silver metal collimator used in normal measurements to reduce scattered radiation contributions (Simon *et al.*, 2003 ▸). In subsequent measurements this was omitted to allow faster acquisition as no significant difference was observed in spectral shape and relative intensities. An all-plastic filter holder was placed directly in front of the detectors for the attenuation measurements which used thin aluminium and copper metal foils, 20 and 45 µm-thick, respectively. Care was taken to avoid metal components, additional scattering and subsequent measurement contamination.

## 3. Results and discussion

**Figure 3:**
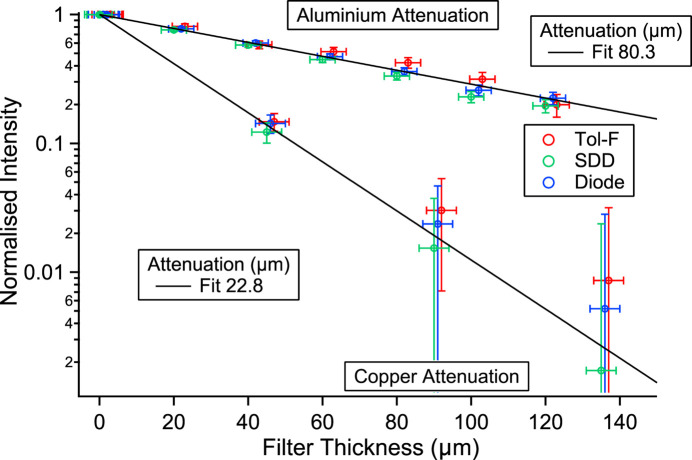
Attenuation of copper *K*-shell fluorescence (SDD), diode and dosimeter (Tol-F) signals with different thickness of aluminium and copper foils. The symbols have been shifted by 1 µm for clarity.

The exact nature of the radiation resulting in the high dose was unclear and the initial dose measurements were inadequate and conflicting. The fluorescence measurements clearly show copper fluorescence but, due to the limited range of the silicon drift detector (2–40 keV), it was not clear whether there was a higher-energy component present in the Tol-F dosimeter (10 keV to 7 MeV; Berthold Tecnologies) measurements as the fluorescence lies below the low energy limit of the measurement range. To clarify this, measurements of the radiation with attenuation by foils of different thickness were made. If the copper fluorescence measurements followed the dosimeter readings then it could be reasonably assumed that the measured radiation was the same and that there was no significant high-energy component present. Fig. 3 ▸ shows the results of the attenuation measurements using the thin foils. Three measurements are shown – integrated copper fluorescence, Tol-F dosimeter and photodiode signals. The measurements have been normalized to the unattenuated signal. All measurements are in good agreement following a similar exponential attenuation with thickness. The solid line for aluminium is calculated for an attenuation length of 80.5 µm, in good agreement with the theoretical value (80.3 µm). For copper foils the experimental points (fit 22.8 µm) are also in good agreement with the expected attenuation for the metal (22.6 µm).

That, in all cases, the measurements closely follow each other confirms that the high dose is due to the copper *K*-shell fluorescence. If a high-energy radiation component was present then the dosimeter reading would remain higher, and the converse for the diode and a low-energy componenent. The Tol-F dosimeter was used for the measurements as its range is greater than that of the LB1236 (30 keV to 1.3 MeV; Berthold Technologies) and more sensitive, the value being two orders of magnitude greater. In addition, the Tol-F has an internal calibration source (Sr-90). The LB1236 is a proportional-counting dosimeter and the Tol-F an ionization-chamber/proportional-counter dosimeter. They also differ in the detector housing/shield – the Tol-F has a thin metal-coated plastic housing and the LB1236 an aluminium casing.

As the radiation is identified as copper *K*-shell fluorescence and not of a higher energy, it clearly originates from the copper mirrors of the visible-light port. Given that the power from 13 mrad of synchrotron radiation for 2.5 GeV electrons and 100 mA beam current is 98 W and that the window transmission at the copper fluorescence (8.05 keV) is 4.2 × 10^−17^, it cannot come from the first mirror: assuming that the conversion efficiency is 100% gives a dose of 1.5 × 10^−5^ µSv h^−1^. The origin of the copper fluorescence and the high dose level of 55 µSv h^−1^ (Tol-F) must be the second mirror, and ionization by the higher-energy scattered radiation transmitted by the quartz vacuum isolation window, which has a cut-off at ∼10 keV.

**Figure 4:**
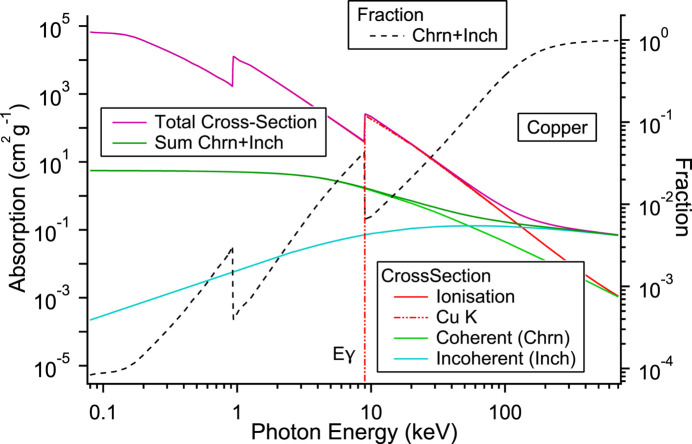
The photoelectron, coherent (Chrn) and Compton (Inch) scattered cross sections for copper are shown (Hubbell *et al.*, 1980 ▸; Chantler *et al.*, 1997,2005 ▸). The fraction of coherent and incoherent scattering of the total cross section is plotted on the right-hand axis.

That such radiation is present is easily seen by the high-energy background in the spectrum of the fluorescence (red curve) and that of the attenuated measurement (green curve) of Fig. 1 ▸. Half of the synchrotron power is emitted above the critical energy of the synchrotron radiation (6.24 keV), approximately 50 W, close to the window cut-off. This power, a substantial amount, is scattered by the first mirror, transmitted further by the UHV window, and ionizes the copper of the second mirror. The resulting fluorescence travels through air into the diagnostic hutch. To compare with the dosimeter reading the scattering needs to be modelled. Such modelling and the calculation of the subsequent radiation dose is frequently carried out by Monte Carlo simulation [*FLUKA* (Ferrari *et al.*, 2005 ▸), *EGS5* (Hirayama *et al.*, 2006 ▸), *PENELOPE* (Salvat & Fernandez-Varea, 2009 ▸), *PHITS* (Sato *et al.*, 2018 ▸)]. Here, due to the setup and the energy of the synchrotron radiation, the scattering from the first mirror responsible for the fluorescence from the second is principally coherent (Rayleigh, Thomson) and not incoherent (Compton), and consequently the calculation can be simplified. For this it is useful to calculate the various quantities using power. The various relevant scattering cross sections (Santra, 2009 ▸; Hubbell *et al.*, 1980 ▸; Chantler *et al.*, 1997,2005 ▸) are shown in Fig. 4 ▸ and plotted as a function of energy. The cross sections are taken from Hubbell *et al.* (1980 ▸) and Chantler *et al.* (1997,2005 ▸).

For photon energies below 100 keV the dominant contribution is the photoelectron ionization cross section. This decreases rapidly with energy and the non-ionizing contributions of the cross sections for coherent and incoherent increase. At energies transmitted by the window, several tens of keV, the other two contributions have a weak but a significant contribution. For high energies the scattering is described by the Klein-Nishina formula which is asymmetric (Santra, 2009 ▸). For the energies of interest here the form is much more Thomson-like with a light asymmetry. For 40 keV photons the fraction of such scattered photons is ∼5% and the asymmetry of the cross section is 15%. As the copper mirror is of polycrystalline nature, the scattering due to diffraction is averaged over angle.

**Figure 5:**
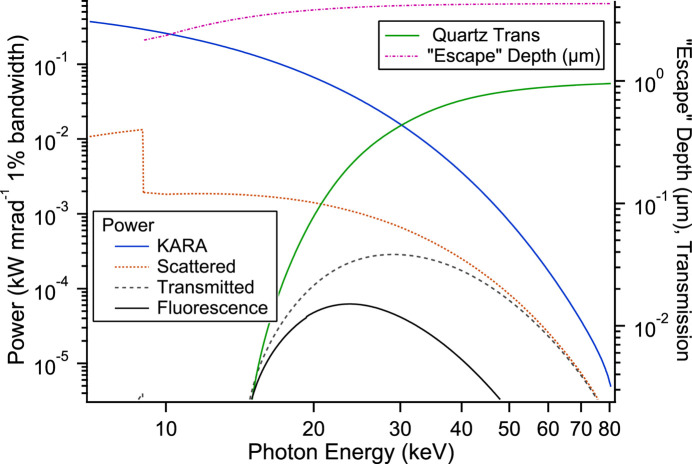
Power from the synchrotron (KARA), scattered by the first mirror (scattered), transmitted by the UHV isolation window (transmitted), and the resulting copper fluorescence from the second mirror (fluorescence). The transmission of the quartz UHV window (quartz trans) and the ‘escape’ depth are plotted on the right-hand axis. Below the copper fluorescence energy of 8.05 keV the ‘escape’ depth has no meaning.

**Figure 6:**
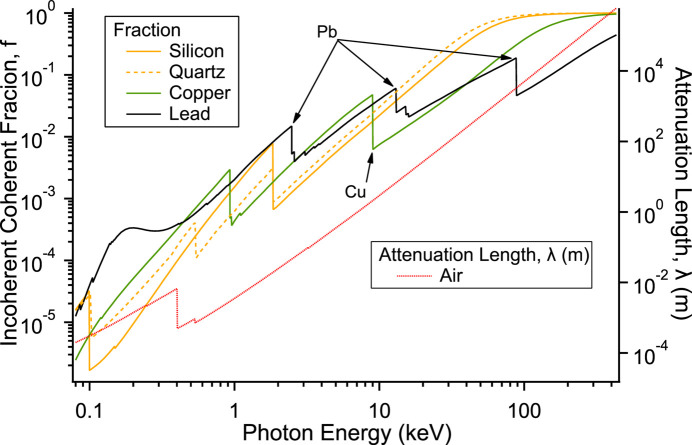
Fraction of coherent and incoherent cross section to the total for silicon, quartz, copper and lead as a function of photon energy. The attenuation length of the photons for air is also plotted on the right-hand axis.

The fraction of photons scattered from the mirror is the sum of the differential scattering cross section and the higher-order multiple-scattering terms to the total cross section: multiple scattering is described by a convolution of the scattering but due to the small value can be neglected apart from the first few terms. The result is shown in Fig. 4 ▸. The distance between the mirrors and their small size is such that the scattering angle and the subtended angle can be considered constant and small, respectively. By using the energy-dependent scattering fraction and the synchrotron power, the power leaving the first mirror is obtained (scattered, Fig. 5 ▸). This, together with the transmission of the 5 mm quartz UHV window (quartz trans, Fig. 5 ▸), determines the transmitted power exiting the window and incident on the second mirror (transmitted, Fig. 5 ▸). It peaks at 25 keV due to the strong absorption of the window and the exponential decay of the synchrotron radiation power with energy [∝ 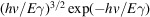 ] above the critical energy *E*γ. The maximum is well above the threshold of the copper *K*-edge, the maximum in the ionization cross section. Whilst the energy is high it is still in the region of a substantial contribution of coherent scattering (see above, and Fig. 4 ▸). The window acts as a low-energy cut-off with a transmission value of 4.2 × 10^−17^ at 8045 eV. Consequently, hardly any copper fluorescence is transmitted by the window. Low-energy silicon fluorescence from the window is also not seen, a consequence of air absorption, additional scattering and the detector geometry (see Fig. 6 ▸).

In addition to blocking a substantial portion of the synchrotron radiation the quartz window blocks high-energy electron and secondary emission, other important ionization contributions. To calculate the dose from the copper fluorescence from the second mirror the ionization and subsequent production of the fluorescence radiation needs to be addressed. This requires an integration over depth of the ionization by the exponentially attenuated incoming photons and the likewise attenuated escape of the fluorescence. The integration gives an effective ‘escape’ depth and is plotted in Fig. 5 ▸. It is energy dependent due to the varying penetration depth of the photons with energy. For high energies the attenuation length of the copper *K*-shell fluorescence limits the ‘escape’ depth, and below the *K*-shell ionization energy has no meaning. Using the photon energy dependent copper *K*-shell ionization cross section, ‘escape’ depth, fluorescence yield and power incident on the second mirror (transmitted) gives the power of the copper fluorescence with photon energy and is plotted (fluorescence) in Fig. 5 ▸. Numerically integrating, with the above solid angles and taking account of absorption due to the air path, gives a value of 5.5 µSv h^−1^ which is in fair agreement with the measured 55 µSv h^−1^ (Tol-F). The attenuation by the thin 30 µm aluminium coating plays little role at the higher copper photon energy and transmits 75% of the fluorescence. The above calculation can be simplified as there is only a small contribution of the Compton scattering in the energy range of the scattered photons: if the maximum energy loss for the scattered photons is assumed, the result differs by 5% demonstrating that there is little Compton scattering present for the scattered photons incident on the second mirror.

Inspecting the spectrum in Fig. 1 ▸ more closely, weak peaks due to additional fluorescence from lead, iron and bismuth are also seen. These peaks are also probably from ionization by the high-energy scattered synchrotron radiation. The mirror has lead housing shielding and also there are other items made of steel in the immediate vicinity. The calculation of these contributions, however, is ill-defined and much more difficult. The remaining high-energy background from the second mirror though can be reasonably modelled. As with the first mirror the coherent and incoherent scattering fraction is used for the scattered photons but in addition the detector response is needed. The result is the purple solid curve in Fig. 1 ▸ and is in good agreement with the unattenuated and attenuated spectra. In addition to the above calculations, simulations using *FLUKA* were also performed. The calculations (Batchelor *et al.*, 2022 ▸) are time consuming and the statistics behind the second mirror and the shielding wall allowed only a dose estimate in the 10 µSv h^−1^ region.

**Figure 7:**
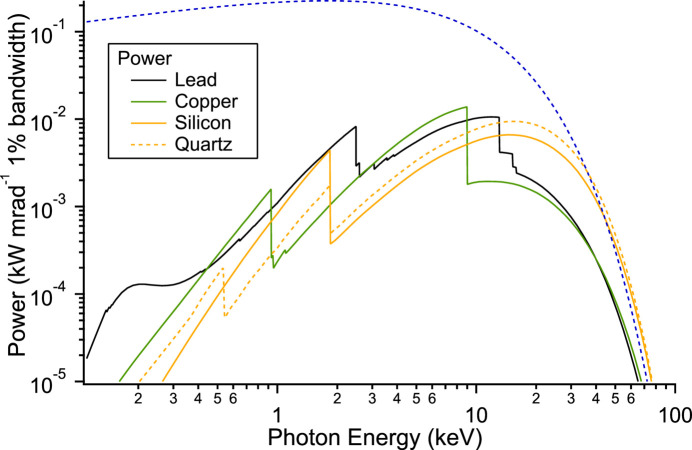
Power of the coherently/incoherently scattered radiation by a single ‘reflection’ (product of KARA power and fraction from Figs. 5 ▸ and 6 ▸) for the different materials. The blue dashed line is the KARA power and is intended as a guide (arbitrarily scaled).

The fractions of coherent and incoherent scattering to the total cross section for silicon, copper, quartz and lead are plotted in Fig. 6 ▸ against photon energy. They are very similar to within an order of magnitude for the different materials, and amount to several percent at energies of tens of keV. Using these fractions and the power of the KARA synchrotron ring the power for a single ‘reflection’ can be calculated and is plotted in Fig. 7 ▸. Considerable power is available to ionize the edges visible in the plot and produce high-energy fluorescence that escapes the material with little attenuation. Silicon has the lowest fluorescence energy but is easily attenuated by an air path (see Fig. 6 ▸). The attenuation length of 20 keV (rough maximum in Fig. 7 ▸) photons in a high atomic number material is only tens of micrometres due to the high photoelectron cross section.

Similarly the coherent scattering cross section is larger for high atomic number compared with low atomic number. Consequently, the radiation is readily ‘reflected’ and escapes or ionizes producing high-energy fluorescence. For silicon the attenuation length is a couple of millimetres and so the material is penetrated further and ionization results in much lower energy fluorescence which is more easily absorbed/attenuated. Glass and silicon with a thin metallic coating are typically used for reflective optics as extensive experience exists in grinding/machining to the desired shape, polishing and covering with a thin metallic reflecting layer. In addition, silicon also has reasonably good thermal conducting properties. The above shows that it is a better choice for reflecting X-ray optics for synchrotrons in comparison with the copper here.

For the optical components of the visible-light port beamline and the energies considered here, coherent scattering dominates and higher-energy Compton scattering, with its associated energy loss, plays a much less significant role allowing a simplification of the calculation. In addition, the scattered radiation only produces copper *K*-shell fluorescence of moderate energy in contrast to higher atomic number material where the higher-energy edges and larger number of decay processes result in higher-energy fluorescence and scattered radiation allowing a build-up of secondary radiation (Asano & Sasamoto, 1994 ▸). Whilst low atomic number material avoids this, it is impractical and a composite of different atomic number materials leads to a more efficient shielding (Hirayama & Shin, 1998 ▸).

## 4. Conclusions

The above experimental results show unequivocally that the higher radiation dose is dominated by the coherently scattered synchrotron background, absorption of which results in copper *K*-shell fluorescence emission from the second mirror. Calculations are able to confirm this both quantitatively (dose) and qualitatively (high photon energy background). The relatively high dose can be avoided by use of silicon or aluminium as mirror material. Finally it is instructive to consider other scenarios. Whilst here we have a quartz window separating the two mirrors and optical components, such a window is not present in X-ray and ultraviolet beamlines and the relatively high dose that can emanate from a beamline relies on additional ‘reflection(s)’, additional shielding and/or a favourable geometry.
